# Site-specific Patient-reported Outcome Measures for Hand Conditions: Systematic Review of Development and Psychometric Properties

**DOI:** 10.1097/GOX.0000000000002256

**Published:** 2019-05-21

**Authors:** Justin C.R. Wormald, Luke Geoghegan, Kyra Sierakowski, Andrew Price, Michele Peters, Abhilash Jain, Jeremy N. Rodrigues

**Affiliations:** From the * Nuffield Department of Orthopaedics, Rheumatology and Musculoskeletal Science (NDORMS), University of Oxford, Oxford, United Kingdom; † Department of Plastic and Reconstructive Surgery, Stoke Mandeville Hospital, Buckinghamshire Healthcare NHS Trust, Aylesbury United Kingdom; ‡ Flinders University, Adelaide, SA, Australia; § Nuffield Department of Population Health, University of Oxford, Oxford, United Kingdom; ¶ Department of Plastic and Reconstructive Surgery, Imperial Healthcare NHS Foundation Trust, London, United Kingdom.

## Abstract

**Background:**

There are a number of site-specific patient-reported outcome measures (PROMs) for hand conditions used in clinical practice and research for assessing the efficacy of surgical and nonsurgical interventions. The most commonly used hand-relevant PROMs are as follows: Disabilities of the Arm, Shoulder and Hand (DASH), QuickDASH (qDASH), Michigan Hand Questionnaire (MHQ), Patient Evaluation Measure (PEM), Upper Extremity Functional Index (UEFI), and Duruoz Hand Index (DHI). There has been no systematic evaluation of the published psychometric properties of these PROMs.

**Methods:**

A PRISMA-compliant systematic review of the development and validation studies of these hand PROMs was prospectively registered in PROSPERO and conducted to assess their psychometric properties. A search strategy was applied to Medline, Embase, PsycINFO, and CINAHL. Abstract screening was performed in duplicate. Assessment of psychometric properties was performed.

**Results:**

The search retrieved 943 articles, of which 54 articles met predefined inclusion criteria. There were 19 studies evaluating DASH, 8 studies evaluating qDASH, 13 studies evaluating MHQ, 5 studies evaluating UEFI, 4 studies evaluating PEM, and 5 studies evaluating DHI. Assessment of content validity, internal consistency, construct validity, reproducibility, responsiveness, floor/ceiling effect, and interpretability for each PROM is described.

**Conclusions:**

The psychometric properties of the most commonly used PROMs in hand research are not adequately described in the published literature. DASH, qDASH, and MHQ have the best-published psychometric properties, though they have either some poor psychometric performance or incompletely studied psychometric properties. There are more limited published data describing the psychometric properties of the UEFI, PEM, and DHI.

## INTRODUCTION

Patient-reported outcome measures (PROMs) are instruments that provide information of the patient’s health status without external interpretation.^1^ PROMs can be generic, domain specific, site specific, or disease specific.^2^ A previous review by our team identified common PROMs used electively managed hand condition research [accepted, in press, *The Journal of Hand Surgery* (Asian-Pacific Volume)]. PROMs demand well-performing psychometric properties in relevant patient populations.^3^ Psychometrics involves the design, deployment, and interpretation of tools to quantify psychological variables, such as questionnaires used to measure hand function or health-related quality of life. Analyses of psychometric properties can be performed after a PROM’s implementation to verify the analyses conducted during development, to investigate unstudied psychometric properties, or to assess its performance under other clinical conditions.

A recent systematic review has assessed the validity and psychometric properties of PROMs used in traumatic hand and wrist conditions but predated the most recent consensus-based psychometric assessment framework.^4,5^ Furthermore, the evidence supporting the validity of the most commonly used PROMs in elective hand conditions has not been assessed against this framework. Prinsen et al^5^ recently published criteria for assessing the quality of published psychometrics. Despite the recency of this publication, the standards by which psychometrics are assessed are well established in the literature. For example, the concept of Rasch analysis has been in circulation since the 1960s.^6^ Evaluation of the published psychometric properties of commonly used hand PROMs, in both elective and traumatic hand conditions, is vital to determine their utility.

The objective of this systematic review is to appraise the psychometric properties of site-specific PROMs commonly used in clinical studies of electively managed and traumatic conditions of the hand against current standards—the Prinsen et al^5^ criteria. The results of this analysis can then be used to assess the suitability of hand-specific PROMs for ongoing use.

## METHODS

The protocol for this systematic review was developed in accordance with the PRISMA statement and prospectively registered in the international prospective register of systematic reviews (PROSPERO) on January 26, 2018 (CRD42018081508). Consensus-based Standards for the selection of health Measurement Instruments (COSMIN) were applied to this systematic review, in accordance with guidance by Prinsen et al.^5^

### Search Strategy

A bespoke search strategy was developed (index and free terms) to identify developmental studies and published psychometric properties of the 6 most commonly used hand site-specific PROMs (Appendix 1). The search strategy was applied in parallel to Medline (1946-February 2018*), Embase (1974-February 2018*), and PsycINFO (1806-February 2018*) provided by the Ovid interface and CINAHL (1981-January 2018*) provided by the NICE Healthcare Databases Advanced Search interface. The search was run on February 15, 2018, and was limited to human studies. The reference list of included articles was hand searched for further relevant publications, and gray literature was searched using Google Scholar at the time of the original literature search. Electronic deduplication was used.

### Eligibility Criteria

**Fig. 1.**
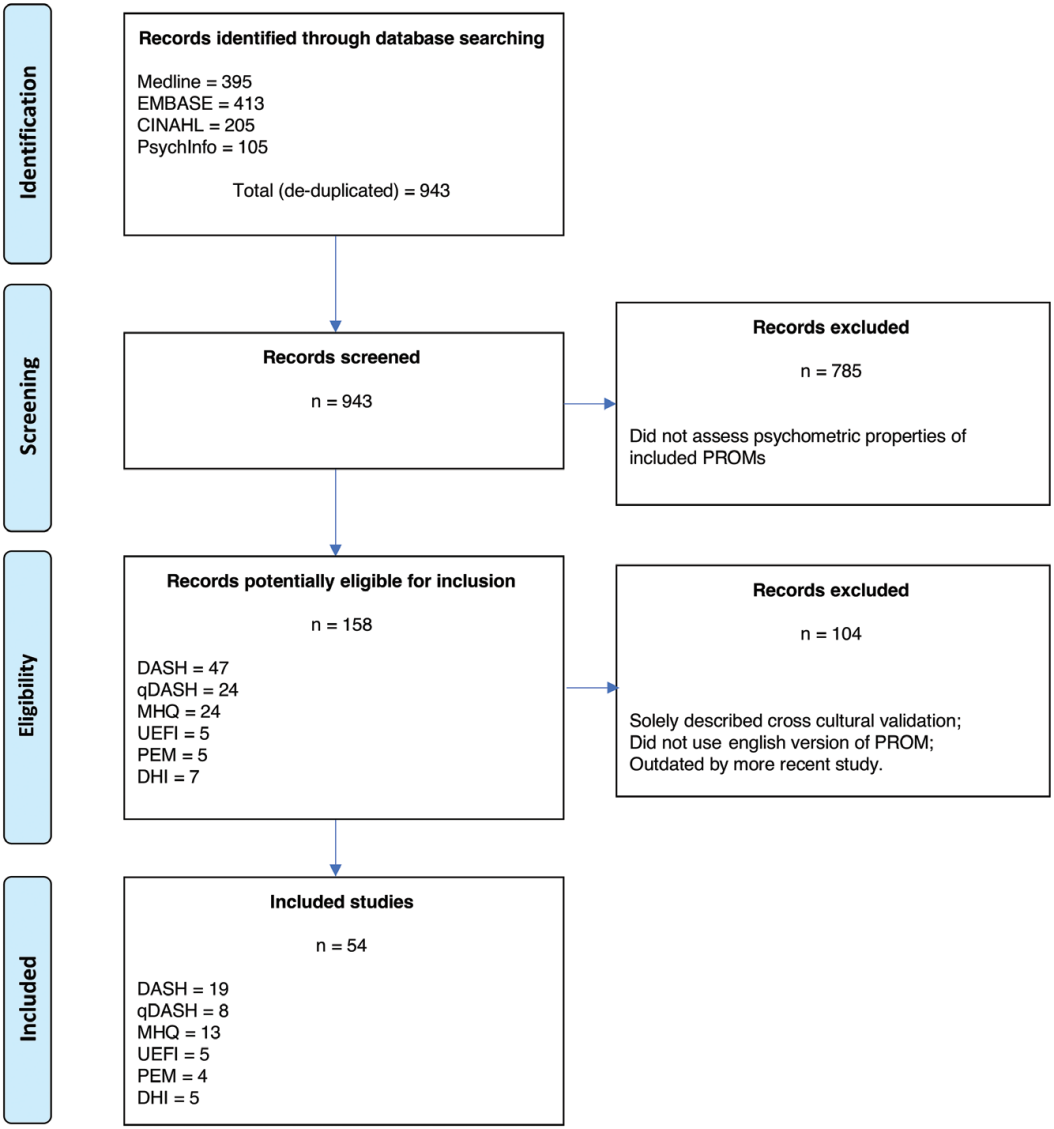
PRISMA flowchart. Adapted with permission from *PLoS Med* 2009;6:e1000097. For more information, visit www.prisma-statement.org.

The PROMs evaluated were those identified in our recent systematic review of elective hand surgery and those identified in the equivalent trauma-based systematic review by Dacombe et al^4^ (excluding wrist-specific PROMs). These were the Disabilities of the Arm, Shoulder and Hand (DASH),^7^ QuickDASH (qDASH),^8^ Michigan Hand Questionnaire (MHQ),^9^ Patient Evaluation Measure (PEM),^10^ Upper Extremity Functional Index (UEFI),^11^ and Duruoz Hand Index (DHI).^12^ All studies of these PROMs in an adult cohort with a condition affecting the hand (distal to the carpal bones) (P) with any applicable intervention and/or comparator (I and C) presenting data related to the psychometric properties of the PROM(s) according to the criteria defined by Prinsen et al^5^ were included. Clinical studies not evaluating one of these PROMs, pediatric studies, and cross-cultural validation studies were excluded. Two authors (JCRW and LG) independently screened all abstracts against a prespecified checklist of criteria for inclusion (Fig. 1). Any disagreement was resolved by consensus discussion and consultation with the third author (JNR) if required.

### Data Extraction

Data extraction was performed in duplicate using a bespoke proforma comprising source, study design, criteria used to assess content validity, internal consistency, criterion validity, construct validity, reproducibility, responsiveness, floor and ceiling effects, and interpretability. Content validity was assessed by the examination of development studies for each PROM. Development methodology was scrutinized for comprehensiveness and relevance based on how the items were generated and selected. Further data regarding study populations in which the PROM was utilized, including patient demographics and disease characteristics, were extracted to determine the generalizability of the study population.

### Data Analysis

A narrative synthesis was utilized to evaluate the published psychometric properties of included PROMs using quality assessment criteria outlined by Prinsen et al.^5^ Specifically, the following properties were assessed for each PROM:

Content validity: the appropriateness to the targeted patient group based on comprehensiveness and relevance.
Structural validity: how well it measures the relevant underlying construct (including Classical Test Theory and Item Response Theory and Rasch measurement theory analyses).
Internal consistency: the extent to which the items within the PROM correlate with each other.
Measurement invariance: whether there are important differences found between group factors (age, sex, and language).
Reliability: the reproducibility of the answers given.
Measurement error: whether smallest detectable change (SDC) in the PROM is smaller than the minimal important change (MIC) and/or minimal important difference.
Construct validity/hypothesis testing: whether it correlates with instruments measuring similar constructs and does not correlate with instruments measuring unrelated constructs.
Criterion validity: whether it correlate with a true “gold standard”, where one exists (typically, this is only applicable when comparing a shortened PROM with its original long form).
Responsiveness: whether the PROM responds to change.

Evidence for each psychometric property was appraised as good performance (+), poor performance (−), indeterminate (?), inappropriate (!), or no evidence (0) to support each psychometric criterion. This was determined by the reviewers and was based on the information across all available studies. “Good performance” ratings denoted that the performance of the PROM across all identified studies of that psychometric measurement property was predominantly good, whereas “poor performance” ratings denoted predominantly poor performance in analyses of that psychometric measurement property in the published data included. “Indeterminate” ratings signified a lack of, or conflicting evidence of, performance, and “inappropriate” ratings were assigned when the measurement property had not been studied using the methods outlined by Prinsen et al.^5^

## RESULTS

**Fig. 2.**
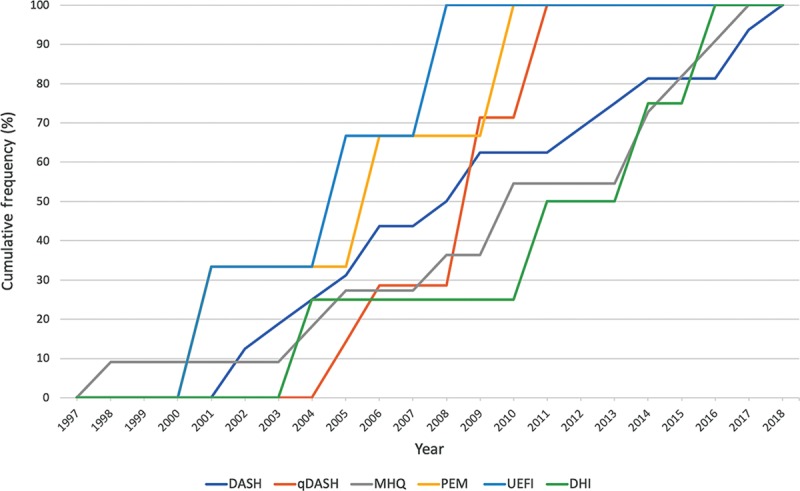
Cumulative frequency of published psychometric evidence over time for included PROMs.

Database searching yielded 943 articles, of which 889 studies were excluded. This left 54 studies, which met predefined inclusion criteria and underwent full analysis (Fig. 1): 19 studies evaluated DASH, 8 studies evaluated qDASH, 13 studies evaluated MHQ, 4 studies evaluated PEM, 5 studies evaluated UEFI, and 5 studies evaluated DHI. Table 1 provides an overview of the reviewed PROMs and populations in which they were validated. Figure 2 demonstrates the cumulative frequency of psychometric evaluation studies for each constituent PROM over time.

**Table 1.**
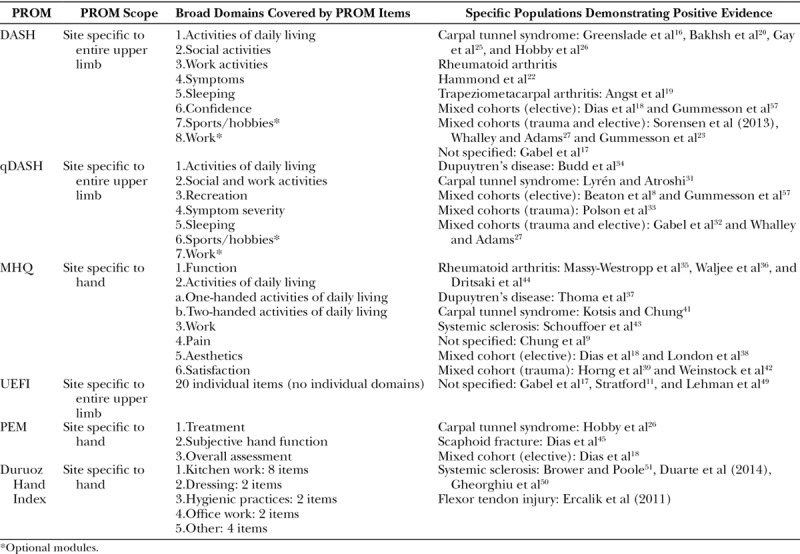
Overview of Included PROMs and Studies

**Table 2.**
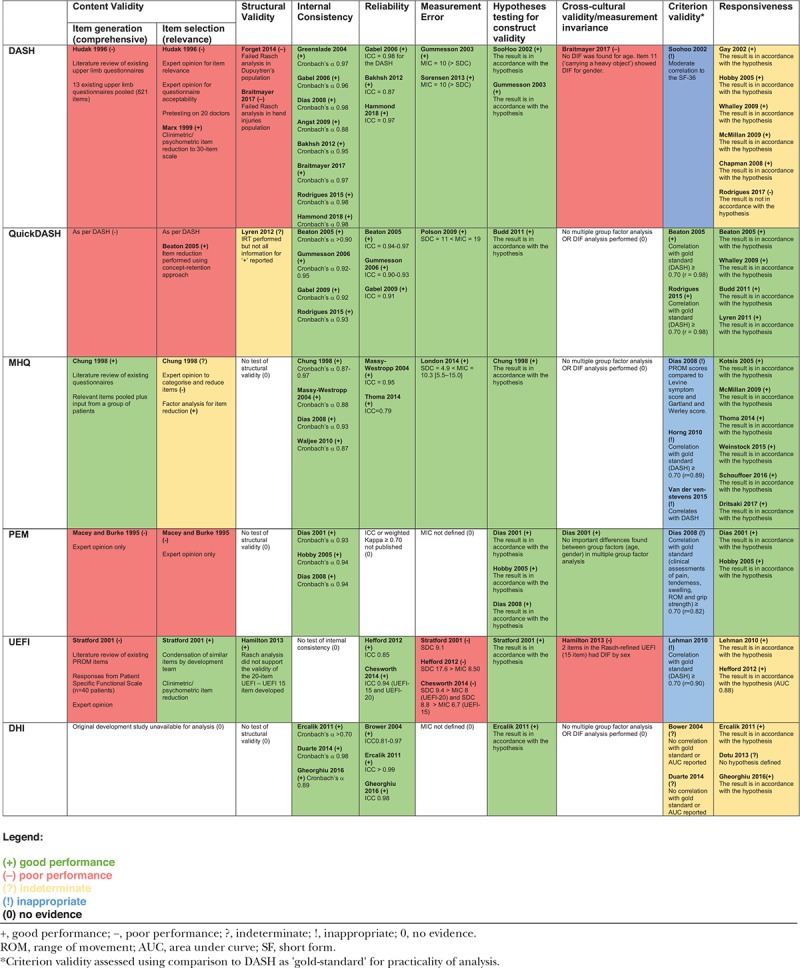
Analysis and grading of published psychometric properties of hand PROMs accoring to the Prinsen criteria.

All PROMs were validated in at least one domain. Carpal tunnel syndrome was the most common pathology in which the psychometric properties had been assessed, but most studies evaluated psychometric properties across mixed cohorts (Table 2). None were validated across all domains. The DASH and the qDASH had the most good performance evidence, determined by the total amount of evidence available across all properties evaluated.

Development studies were available for analysis for the DASH, qDASH, MHQ, PEM, and UEFI. Only the MHQ explicitly sought information from a group of patients in item generation. DASH, MHQ, and UEFI were based on literature reviews of existing upper limb PROMs, whereas the PEM was developed by expert opinion only. Item selection was performed using a combination of expert opinion and clinimetric/psychometric item reduction for the DASH, MHQ, and UEFI and for the conversion of the DASH to the qDASH. The development studies for the DHI were not available despite email request.

The DASH had the most published research assessing structural validity. These studies demonstrated poor performance for factor analysis and item response theory (IST) analyses. The qDASH used Rasch modeling in its development, along with 2 other methods of item reduction but has not undergone formal structural validity assessment. The original 20-item UEFI failed Rasch measurement model and so was refined into a 15-item questionnaire, which fitted a Rasch model. All other PROMs had no published evidence for or against their structural validity.

All included PROMs had supporting evidence of internal consistency with values for Cronbach’s *α* ranging from 0.87 to 0.98, with many showing evidence of redundancy of items (Cronbach’s *α* >0.90).

Almost all of the included PROMs had evidence of reliability with intraclass correlation coefficients (ICCs) >0.80 apart from the PEM, where no reliability studies were found. The DASH, qDASH, and MHQ had published MICs that were larger than the SDC of the PROM. The PEM and DHI did not have published MICs that could be identified. The UEFI has 2 studies published that show an SDC greater than the MIC, indicating potential for measurement error.

All PROMs in the analysis had evidence of construct validity when they were compared with similar instruments.

The DASH, UEFI, and PEM underwent formal testing of measurement invariance. Differential item functioning (DIF) found sex-related issues in the DASH and UEFI for task-specific items. Multiple group factor analysis for PEM showed no differences between age and sex for its items.

Criterion validity was often inappropriately assessed. The only appropriate analysis possible in this review’s scope was to assess the qDASH against the DASH, which had been done, and demonstrated good performance. Studies evaluating against inappropriate gold standards were present for MHQ, PEM, and UEFI.

Almost all of the PROMs in the analysis had published evidence of responsiveness to change in the tested populations. There were some discrepancies in the published evidence depending on the populations. For example, DASH was not responsive to change in patients with Dupuytren’s disease but was in populations with other hand conditions.

## DISCUSSION

This systematic review has evaluated the psychometric properties of commonly used PROMs in hand conditions using internationally accepted, consensus-based criteria. The psychometric properties of the 6 included PROMs were largely only studied incompletely. In some cases, poor psychometric performance was identified, with no single outcome measure demonstrating sufficient psychometric robustness to meet the Prinsen et al^5^ criteria.

### Content Validity

Modern development of a PROM requires extensive, multicenter, and multinational data collection exercises to ensure a comprehensive list of items is generated, applicable to different populations and cultures. Alongside primary data collection, systematic reviews of existing PROMs are required to ensure comprehensive inclusion of all relevant items. Following this, item reduction should be performed through field testing and psychometric statistical techniques, including the use of item response theory and Rasch modeling. For some PROMs (DASH, qDASH, MHQ, and UEFI), psychometric analyses were used to reduce the items to the current questionnaires, whereas the PEM employed expert opinion alone. The most commonly used hand PROMs, therefore, do not fully meet contemporary standards for PROM development, particularly in the item generation process, raising questions about their comprehensiveness.

### Structural Validity

Only 2 PROMs had evidence of structural validity testing: the DASH and the UEFI. Development of the qDASH involved Rasch modeling for item reduction, but we could not identify the evidence of structural validity testing specifically.^8^ Forget et al^14^ demonstrated that the DASH had large ceiling effects and failed to meet the assumptions of the Rasch measurement model in a population of patients with Dupuytren’s disease. Additionally, the original UEFI 20-item questionnaire did not fit a Rasch model in a population of undefined upper extremity conditions. It is important to note that whereas the DASH and UEFI performed poorly when subjected to Rasch modeling, it is unknown to what extent the other PROMs in this review would perform. There is, therefore, a risk of disproportionate criticism of the DASH and UEFI. Based on structural validity alone, the choice of a hand PROM is currently between those with known suboptimal performance and others with unknown performance, which may be the same, better, or worse.

This review only included PROMs that have been deployed in clinical studies, which was used as a benchmark of practical usefulness of the PROMs. There are other PROMs that have been recently developed, such as the PROM Information System Upper Extremity (PROMIS UE) tool.^53^ The PROMIS UE is similar to the DASH in that it is site specific to the upper limb and correlates well with the qDASH.^54^ It has been developed using item response theory and is a more complex, accurate, and dynamic tool.^53^ Furthermore, it can be delivering using computer adaptive testing, which improves its deployment and utility across digital platforms.^55^ This is an encouraging development, though it was not analyzed here as its application is yet to be confirmed in clinical studies. Based on this review, further study of the structural validity of existing PROMs is an important area for future research in this field.

### Internal Consistency

Internal consistency was evaluated in all PROMs, each of which demonstrated positive ratings with Cronbach’s *α* scores ranging from 0.87 to 0.98, suggesting high interrelatedness among constituent outcome measure items. Although an *α* of >0.70 is considered to be desirable in the framework used,^5^ other sources suggest that very high values, >0.90, are indicative of redundancy of items within the PROM and are not desirable.^45^

### Measurement Invariance

Only 3 studies reported multiple group factor analysis. Measurement invariance assesses the equivalence of items across specified groups, with only one study reporting no significant differences between group factors (sex, hand dominance, and side injured) and PEM score.^45^ Braitmayer et al^15^ conducted multifactor analysis for the DASH and reported DIF for sex. The Rasch-refined UEFI showed DIF for sex on 2 items “using tools/appliances” and “cleaning”.^46^ The PEM comprises symptom items (pain, stiffness, etc), whereas the DASH items with pronounced DIF were task based, specifically item 11 (carrying a heavy object) demonstrated important differences between sex,^15^ which was mirrored by the task-based items with DIF in the UEFI.^46^ The site-specific PROMs appraised here comprise some with mainly task-based items, some with symptom-based items, and some that are a mix of the 2.

### Reliability

Reliability was frequently reported and rated positively across all PROMs except from the PEM, where the intraclass correlation coefficient or weighted Kappa was not reported. This was one of the better assessed aspects of psychometric performance across all PROMs.

### Measurement Error

There was limited evidence of measurement error assessment, where the MIC is assessed relative to the SDC. This requires the MIC to be calculated. MIC is one element of the interpretability of a PROM, and interpretability is not central in the COSMIN system. MICs have been reported for 4 of the included PROMs (DASH, qDASH, MHQ, and UEFI),^21^ and these were the PROMs with measurement error studies. For the UEFI, the SDC was reported as 8.8–17.6 with an MIC as 6.7–8.5 across 3 studies. This brings into question the ability of the UEFI to accurately measure the minimal clinically important change in the populations it has been tested in.

### Criterion Validity

Criterion validity was inappropriately studied in the majority of PROMs, principally due to the lack of a gold standard for valid comparison to be made. Most studies reported correlation with the DASH as a gold standard instrument, which implies inappropriately that the DASH is a perfect outcome measure. Criterion validity is accepted in the comparison of shortened versions of PROMs to their longer constituents, and this was reported in the comparison of qDASH and DASH.^8^

### Construct Validity

This property was widely studied, with all PROMs evaluated and demonstrating positive supporting evidence. The PROMs studied were frequently compared with generic quality of life measures such as the Short-Form 12 and Short-Form 36 to examine the behavior of the instrument in relation to the underlying concept it designed to measure. There are similarities between “construct validity” hypothesis testing the relationship between the PROM being studied and other measures, and the criterion validity studies criticized already. The distinction made here was that appropriate hypothesis testing looked for convergent or divergent validity by comparing with multiple similar measures, and/or seemingly unrelated measures.

### Responsiveness

Several studies have reported that the DASH is a responsive instrument in cohorts of patients with carpal tunnel syndrome^25,26^ and arthritis.^27^ However, Rodrigues et al^30^ examined the responsiveness of the DASH in patients with Dupuytren’s disease following fasciectomy and dermofasciectomy and found that it could not distinguish patients who had experienced meaningful change in hand function following intervention. Consequently, the DASH was found to have moderate responsiveness and poor interpretability in patients with Dupuytren’s disease, meaning that an MIC could not be estimated. Conflicting evidence regarding the responsiveness of the DASH may be due to the fact that clinically significant improvement is not always made following intervention. Moreover, as a site-specific PROM for the entire upper limb, the ability of the instrument to discern changes in specific functional aspects of the hand and digits is questionable. In contrast, the MHQ, a hand-specific PROM, was found to have a high effect size when used in a cohort of patients following fasciectomy for Dupuytren’s disease, indicating that the items in the MHQ are sensitive to change in hand function following intervention.^37^

### Limitations

The findings of this review make it difficult to recommend the most suitable outcome measure at present. Indeed, the only core outcome set for hand surgery listed by the Core Outcome Measures in Effectiveness Trials initiative suggests combining the use of site-specific and disease-specific outcome measures in Dupuytren’s disease but does not provide further detail.^56^ Despite a robust search strategy, relevant publications may not have been identified from our original search. This review used only one framework for analysis.^5^

## CONCLUSIONS

In summary, the results of this systematic review indicate that currently implemented PROMs have incomplete evidence to support their use in hand surgery research and practice, when compared with contemporary PROM standards (Appendix, http://links.lww.com/PRSGO/B116). The DASH, qDASH, and MHQ have the most published data evaluating their psychometric properties but have shortcomings and evidence gaps based on this review. There was more incomplete evidence to support the psychometric properties of the UEFI, PEM, and DHI for use in hand surgery practice and research. Future research in hand conditions could consider the role of contemporary PROMs, particularly those that have been developed using IRT, as such PROMs are more likely to have evidence that they meet modern psychometric standards.
